# Nasal colonization and antimicrobial susceptibility pattern of *Staphylococcus aureus* among pre-school children in Ethiopia

**DOI:** 10.1186/s13104-017-3079-6

**Published:** 2017-12-19

**Authors:** Alemayehu Reta, Moges Wubie, Getnet Mekuria

**Affiliations:** 1 Department of Medical Laboratory Science, College of Health Sciences, Debre Markos University, Pobox-269, Debre Markos, Ethiopia; 2 Department of Public Health, College of Health Sciences, Debre Markos University, Debre Markos, Ethiopia

**Keywords:** Antimicrobial susceptibility pattern, *Staphylococcus aureus*, Nasal colonization, Pre-school children

## Abstract

**Background:**

*Staphylococcus aureus* is one of the bacterium that can asymptomatically colonize the human upper respiratory tract (i.e. nose and throat). Carriage of *S*. *aureus*, including methicillin resistant *S. aureus*, is common to children. The aim at this study was to determine the nasal colonization, associated factors and antimicrobial susceptibility patterns of *S. aureus* isolates among pre-school children in Debre Markos town.

**Methods:**

Institutional-based cross sectional study was conducted. A total of 400 nasal swabs were collected from pre-school children from April to June, 2015 following standard microbiological methods. MRSA was detected using both Cefoxitin (30 μg) and Oxacillin (6 μg) (Oxoid Ltd. England) discs in combination and associated factors were assessed using self-administered pretested questionnaires, which were delivered to the children’s parents/guardians. Statistical analysis of the data (logistic regression) was done using SPSS V-22.

**Results:**

A total of 52 *S. aureus* isolate was recovered from 400 nasal swap samples. The prevalence of *S. aureus* among pre-School children was 13% (52/400). The susceptibility patterns of the isolates to commonly used antibiotics were: 84.62% to Chloramphenicol, 69.2% to Doxycycline and Tetracycline, 92.3% to Kanamycin, 7.7% to Ampicillin and Penicillin, 86.6% to Ceftriaxone, and 76.9% to Augmentin. All the isolates were sensitive to Oxacillin and Cefoxitin, mean there was no methicillin resistant *S. aureus* isolate, and also sensitive to Gentamycin, Erythromycin and Clindamycin. The main associated factors of nasal colonization of *S. aureus* in the study area was, having recurrent acute otitis media (AOM) [AOR = 2.37 (1.11, 5.06)], Children admission in hospital [AOR = 1.96 (1.03, 3.73)] and cough [AOR = 2.09 (1.08, 4.09)].

**Conclusions:**

The prevalence of *S. aureus* nasal colonization among pre-school children was relatively low in absence of MRSA. Factors like; recurrent AOM, hospital admission and cough were significantly associated with *S. aureus* nasal colonization. Most of the isolates were resistant to β-lactam drugs and sensitive to drugs like Gentamycin, Erythromycin, Clindamycin, Chloramphenicol, Doxycycline, Tetracycline, Kanamycin and Augmentin.

## Background

Nasal colonization of *S. aureus* creates a risk factor of developing invasive disease. Children are commonly considered to be persistent carriers more frequently than adults, with the highest rate of carriage reached in the first year of life and transition from persistent to intermittent or non-carriage occurring during adolescence [1].

There is increasing evidence that *S. aureus* nasal colonization is spreading among healthy pre-school children. Studies conducted around the world reveal that, 17.5% in Japan [2], 34% in Belgium [3], 35% in India [4] and Republic of Korea [5], 48% in Brazil [6], and 17.3% in Turkey [7] of pre-school children were colonized by *S. aureus*, even if the associated factors are not readily identified. While some studies have demonstrated recurrent acute otitis media (AOM), frequent exposure to antibiotics [8], socioeconomic status [9], healthcare worker in the home [10], child-care attendance [11], hospitalization, chronic illness and previous isolation of methicillin resistant *S. aureus* (MRSA) [12] but there is no definite consensus as to why healthy children acquire these resistant bacteria [9]. There are few studies conducted to assess nasal colonization of *S. aureus* among pre-school children around the globe and to our best knowledge, there is no study conducted on factors associated with nasal colonization of *S. aureus* particularly among pre-school children in the study area and in Ethiopia at large. Therefore this study will create a better understanding of the epidemiology and determinants of *S. aureus* nasal colonization could positively be helpful in improving infection control and prevention strategies and designing protocols.

## Methods

### Study enrollment, specimen collection and processing

Institution based cross sectional study was conducted at kindergarten school from April to June, 2015. All sampled pre-school children (1–6 years) who were attending their kindergarten education in Debre Markos town during the study period were selected by using simple random sampling technique. Children were excluded from the study, if they had received antibiotic treatment for the previous 7 days. Individual variables were obtained from the children’s guardians/parents immediately after written consent is obtained from the guardians/parents and before swabs were collected. After written informed consent was obtained, each child had a specimen collected from the anterior nares with a dry, sterile and moistened swab (Ventura Tran system; Copan Diagnostics, Corona, Calif.). The tip of the collection swab was inserted 2–3 cm into the anterior nares and rotated 4–5 times both clockwise and anticlockwise before withdrawal in each nostril. Swabs were placed into Screw capped test tubes containing enrichment broth (contained 37.5 g sodium chloride (NaCl), 1.25 g yeast extract, 5.0 g tryptone, and 500 ml distilled water) and the samples were labeled, packaged and transported to the Medical Microbiology Laboratory of Debre Markos University within 2 h of collection and processed immediately according to standard Microbiological procedures [13].

### Selective media and culture conditions

Each 10 μl of incubated enrichment broth was inoculated in both blood agar and MSA (Oxoid Ltd., England) and incubated at 35 °C for 24–48 h. Golden yellow colonies of the MSA and white creamy colonies in blood agar indicate *S. aureus*, which was subsequently identified by gram staining, 3% catalase testing, tube coagulase testing with the Staph Latex agglutination assay (Life Sign, Somerset, N.J.) [14].

### Identification of MRSA

*Staphylococcus aureus* isolates were tested for Methicillin resistance by using modified Kirby-Bauer disc diffusion technique [15]. Inocula were adjusted to 0.5% McFarland standard and each streaked uniformly with the swab sticks in Mueller–Hinton agar plates containing 6.5% NaCl to obtain confluent growth [16]. The plates were allowed to dry for 3–5 min before the disks used. The Oxacillin (6 µg) and Cefoxitin (30 µg) discs were used for susceptibility testing. The plates then incubated aerobically at 35 °C for 24 h. The results were interpreted according to Clinical and Laboratory Standard Institute (CLSI) guidelines for Oxacillin and Cefoxitin susceptibility testing: a zone size of < 10 mm was considered resistant; a zone size of > 13 mm was considered susceptible for Oxacillin and a zone size of < 21 mm was considered resistant; a zone size of > 22 mm was considered susceptible for Cefoxitin [17].

### Antibiotic susceptibility testing

The antibiotic susceptibility testing was performed by modified Kirby-Bauer disc diffusion technique and MIC for Ceftriaxone [15]. The colonies were picked up with wooden applicator stick and dipped into nutrient broth to make direct colony suspension of the isolates and inocula were adjusted at 0.5% McFarland standard. After few minutes these suspensions were streaked with Mueller–Hinton agar plates. The antibiotic susceptibility testing was performed on the following antibiotic discs; Ceftriaxone (30 μg), Chloramphenicol (30 μg), Clindamycin (2 μg), Erythromycin (15 μg), Gentamicin (10 μg), Penicillin G (10 units), Doxycycline (30 μg), Tetracycline (30 μg), Kanamycin (30 μg), Amoxicillin–Clavulanic acid (20 μg), Cefoxitin (30 µg), Ampicillin (10 μg) and Oxacillin (6 μg). All antibiotic discs/drugs were obtained from Oxoid Ltd. England. The plates were incubated at 37 °C for 18–24 h.

The disc diffusion technique and zone interpretation of each antimicrobial agent were used in accordance with CLSI guidelines [16]. Diameters of the zone of inhibition around the disc were measured using a graduated caliper in millimeters and the isolates was classified as sensitive, intermediate, and resistant according to the standardized table supplied by the CLSI. *Staphylococcus aureus* (ATCC-25923) was used as control for the antimicrobial susceptibility pattern.

### Data analysis

The collected data were checked for completeness, coded, and then entered into Epi data version 3.1 software for an edition and cleaning of data and export to SPSS version 22 Software for analysis. Bivariate and multivariate logistic regression analysis were performed to identify factors associated with nasal colonization of *S. aureus*.

## Results

### Socio-demographic characteristics

Of the 422 eligible parents/guardians, 400 agreed to participate in the study, which made a response rate of 94.8%. The median age of pre-school children were 5 years (Inter quartile range, IQR ± 3). With regard to household monthly income, the median income were 3100 birr (Inter quartile range, IQR ± 16, 267). More than half (55%) of households had income of 2001–5000 birr monthly (Table 1).

**Table 1:** Socio-demographic characteristics of pre-school children, Debre Markos, North Ethiopia, 2015

Demographic characteristics (n = 400)	No (%)
Sex of child	
Male	160 (40.0)
Female	240 (60.0)
Age of child (years)	
≤ 4	169 (42.3)
> 4	231 (57.8)
Younger sibling	
0	240 (60.0)
1–3	160 (40.0)
Older sibling	
0	136 (34.0)
1–3	264 (66.0)
Educational status of mother	
Unable to read & write	32 (8.0)
Primary education	72 (18.0)
Secondary education	152 (38.0)
College & above	144 (36.0)
Household monthly income^a^	
400–2000	112 (28.0)
2001–5000	220 (55.0)
5001–16,667	68 (17.0)

^a^Ethiopian Birr

### Prevalence of *S. aureus*

About 74% (296/400) of the pre-school children involved in the study were aged > 4 years, of these 12% (48/400) were colonized by S*. aureus*. Majority of the study participants were females, which accounts 60% (240/400) among them 8% (32/400) were colonized by *S. aureus*. The overall prevalence of *S. aureus* were 13% (52/400) (Table 2). The good news from our study was, there was no MRSA isolates among *S. aureus* colonized pre-school children.

**Table 2:** Age and sex distribution of *S. aureus* colonization among pre-school children Debre Markos town, 2015

Characteristics	*S. aureus* colonization		Total
	Yes	No	
Age			
Median	6	5	5
Range	3	3	3
Age (years)			
> 4	48 (12%)	248 (62%)	296 (74%)
≤ 4	4 (1%)	100 (25%)	104 (26%)
Total	52 (13%)	348 (87%)	400 (100%)
Sex			
Male	20 (5%)	140 (35%)	160 (40%)
Female	32 (8%)	208 (52%)	240 (60%)
Total	52 (13%)	348 (87%)	400 (100%)

### Medical and related characteristics

The majority (87.8%) of pre- school children didn’t colonize by *S. aureus*. Fifty-seven (14.3%) of pre-school children had recurrent AOM. More than half (52.8%) of pre-school children had Cough in the previous 30 days. One-fourth (25.3%) of pre-school children was admitted to hospital in the previous 12 months (Table 3).

**Table 3:** Medical and related characteristics of pre-school children, Debre Markos, North Ethiopia, 2015

Characteristics	No (%) (n = 400)
Presence of *S. aureus* (child)	49 (12.3)
Skin/soft tissue infection (child)	107 (26.8)
Having recurrent acute otitis media (child)	57 (14.3)
Cough^a^ (child)	211 (52.8)
Child admitted to hospital^b^	101 (25.3)
Child use of antibiotic^b^	201 (50.3)
Use of antibiotic by a child of the household^b^	197 (49.3)
Use of antibiotic by a family member^b^	180 (45.0)
Hospitalization of family member^b^	52 (13.0)
Household member contact with health institution workers	120 (30.0)
Family member with wound^b^	94 (23.5)

^a^In the previous 30 days

^b^In the previous 12 months

### Factors associated with nasal colonization of *S. aureus*

Hosmer–Lemeshow goodness-of-fit tested (p = 0.425) was used to assess the fitness of the model. During the bivariate logistic regression analysis, child admission to hospital in the previous 12 months, Skin/soft tissue infection, having recurrent AOM, cough in the previous 30 days, family member with wound in the previous 12 months and child use of antibiotic in the previous 12 months were significantly associated with nasal colonization of *S. aureus*.

During the multivariate logistic regression analysis, child admission to hospital in the previous 12 months, having recurrent AOM and cough in the previous 30 days was significantly associated with nasal colonization of *S. aureus.*

Children who were admitted in hospital in the previous 12 months were 1.96 times more likely colonized by *S. aureus* than those children not admitted [AOR = 1.96 (1.03,3.73)]. Those children that had recurrent AOM were 2.37 times more likely colonized by *S. aureus* than children hadn’t recurrent AOM [AOR = 2.37 (1.11,5.06)]. Those children that had coughed in the previous 30 days were 2.09 times more likely colonized by *S. aureus* than children hadn’t coughed [AOR = 2.09 (1.08,4.09)] (Table 4).

**Table 4:** Bivariate and multivariate logistic regression analysis showing factors associated with nasal colonization by *S. aureus* among pre-school children, Debre Markos, North Ethiopia, 2015

Variable	Presence of *S. aureus*		Crude odds ratio (95% CI)	Adjusted odds ratio (95% CI)	*P* value
	No (%)	Yes (%)			
Child admitted to hospital in the previous 12 months					
Yes	20 (5.0)	81 (20.2)	2.29 (1.24, 4.28)	1.96 (1.03, 3.73)	0.040
No	29 (7.2)	270 (67.5)	1	1	
Skin/soft tissue infection					
Yes	19 (4.8)	88 (22.0)	1.89 (1.02, 3.53)		
No	30 (7.5)	263 (65.8)	1		
Having recurrent AOM					
Yes	12 (3.0)	45 (11.2)	2.21 (1.07, 4.54)	2.37 (1.11, 5.06)	0.026
No	37 (9.2)	306 (76.5)	1	1	
Cough in the previous 30 days					
Yes	34 (8.5)	177 (44.2)	2.23 (1.17, 4.24)	2.09 (1.08, 4.09)	0.029
No	15 (3.8)	174 (43.5)	1	1	
Family member with wound in the previous 12 months					
Yes	18 (4.5)	76 (19.0)	2.10 (1.12, 3.96)		
No	31 (7.8)	275 (68.8)	1		
Child use of antibiotic in the previous 12 months					
Yes	18 (4.5)	183 (45.8)	1.88 (1.01, 3.48)		
No	31 (7.8)	168 (42.0)	1		

Backward stepwise logistic regression

### Evaluation of antimicrobial susceptibility tests

The in vitro antimicrobial susceptibility pattern results in 52 *S. aureus* isolates were shown in Table 2. A total of 52 *S. aureus* isolates were subjected to antibiotic susceptibility test against 13 antimicrobial drugs (Table 5). The susceptibility patterns of the isolates to commonly used antibiotics were: 84.62% to Chloramphenicol, 69.2% to Doxycycline and Tetracycline, 92.3% to Kanamycin, 7.7% to Ampicillin and Penicillin, 86.6% to Ceftriaxone (but after performing minimum inhibitory concentration (MIC), all the isolates become susceptible for Ceftriaxone), and 76.9% to Augmentin. All the isolates were sensitive to Oxacillin and Cefoxitin, mean there was no MRSA isolate and also sensitive to Gentamycin, Erythromycin and Clindamycin.

**Table 5:** Antimicrobial susceptibility patterns of *S. aureus* isolates, Debre Markos town, 2015

Antimicrobial drugs	*S. aureus* isolates susceptibility pattern, N = 52		
	Sensitive no (%)	Intermediate no (%)	Resistant no (%)
Penicillin	4 (7.7)	0 (0)	48 (92.3)
Ampicillin	4 (7.7)	0 (0)	48 (92.3)
Oxacillin	52 (100)	0 (0)	0 (0)
Cefoxitin	52 (100)	0 (0)	0 (0)
Augmentin	40 (76.9)	0 (0)	12 (23.1)
Ceftriaxone	52 (100)	0 (0)	0 (0)
Gentamycin	52 (100)	0 (0)	0 (0)
Kanamycin	48 (92.3)	4 (7.7)	0 (0)
Erythromycin	52 (100)	0 (0)	0 (0)
Tetracycline	36 (69.2)	0 (0)	16 (30.8)
Doxycycline	36 (69.2)	4 (7.7)	12 (23.1)
Clindamycin	52 (100)	0 (0)	0 (0)
Chloramphenicol	44 (84.62)	0 (0)	8 (15.38)

### Drug resistance pattern/antibiogram of *S. aureus*

The resistance patterns of *S. aureus* isolates varied from one to six antimicrobial drugs. The highest resistance pattern (46.15%) was observed for two antibiotics with pattern of Penicillin/Ampicillin. Three resistance patterns of equal proportion were observed for resistance to one, three and six antibiotics such as Chloramphenicol, Penicillin/Ampicillin/Tetracycline and Penicillin/Ampicillin/Augmentin/Tetracycline/Doxycycline/Chloramphenicol, respectively and also Penicillin/Ampicillin/Augmentin and Penicillin/Ampicillin/Tetracycline/Doxycycline had the same resistance pattern proportion. Maximum resistance (resistant to six drugs) was observed for one *S. aureus* isolate from resistance pattern of Penicillin/Ampicillin/Augmentin/Tetracycline/Doxycycline/Chloramphenicol. Multi-drug resistance (defined as resistance to at least three different antibiotic groups) *S. aureus* isolates were observed (Table 6).

**Table 6:** Drug resistance pattern of *S. aureus* isolates for different antibiotics, Debre Markos town, 2015

No of antimicrobial drugs	Resistance pattern	Resistant isolates, No (%)
1	Chl	4 (7.69)
2	Pen/Amp	24 (46.15)
3	Pen/Amp/Aug	8 (15.38)
	Pen/Amp/Tet	4 (7.69)
4	Pen/Amp/Tet/Dox	8 (15.38)
6	Pen/Amp/Aug/Tet/Dox/Chl	4 (7.69)

*Pen* penicillin, *Amp* ampicillin, *Aug* augmentin, *Tet* tetracycline, *Dox* doxycycline, *Chl* chloramphenicol

## Discussion

In the present study, the overall frequency of isolation of *S. aureus* from pre-school children of Debre Markos town was 13% (52/400). In contrast with this, a study conducted in Belgium, [3], around 50% of pre-school children were colonized with *S. aureus*. This study also performed a prospective cohort study, how socio-economic status affects *S. aureus* nasal colonization among preschool children, and conclude that there is high *S. aureus* nasal colonization among preschool children that have high socio-economic status when we compare with low socio-economic status. As we know our country, Ethiopia is one of a developing country having low socio-economic status. This factor may be the reason why the current study obtained decreased *S. aureus* nasal colonization among pre-school children in Debre Markos town. In addition to this, seasonal variations, geographical distribution and method of isolation may affect the prevalence.

In line with the current study, a study conducted in Japan, [2], the prevalence of *S. aureus* nasal colonization among children attending day care center having 1 month–5 year ages was 17.9% and also a study conducted in Turkey, [7] reveals that, 17.3% of pre-school children were colonized by *S. aureus.*

The susceptibility patterns of the isolates to commonly used antibiotics were: 84.62% to Chloramphenicol, 69.2% to Doxycycline and Tetracycline, 92.3% to Kanamycin, 7.7% to Ampicillin and Penicillin, 86.6% to Ceftriaxone, and 76.9% to Augmentin. All the isolates were sensitive to Oxacillin, Gentamycin, Erythromycin and Clindamycin. The findings of our current study were similar with a study conducted in Turkey [7], all *S. aureus* isolates were sensitive to Gentamycin, Erythromycin and Clindamycin.

Our finding showed that, there is no methicillin resistant *S. aureus* isolates. This finding is in agreement with a study conducted in Turkey, [7], they isolated only one MRSA (0.1%) from a newborn infant that had been hospitalized during the first week of his life in the neonatal intensive care unit, which may suggest hospital acquired/nosocomial origin.

The prevalence of carriage and the effect of various epidemiological factors of nasal colonization of *S. aureus* has been described, but results vary from one study to another, in parallel with differences in the populations included or definitions used.

A study conducted in India shows that, attending kindergarten schools or being in pre-school are one factor to be colonized with *S. aureus* [4]. Through such investigation these pre-school children could be target groups to undergo further assessment on factors related to nasal colonization of pre-school children with *S. aureus*. In our current study, the main associated factors of nasal colonization of *S. aureus* among pre-school children was Child admitted to hospital in the previous 12 months [AOR = 1.96 (1.03, 3.73); p = 0.040], having recurrent AOM [AOR = 2.37 (1.11, 5.06); p = 0.026] and Cough in the previous 30 days [AOR = 2.09 (1.08, 4.09); p = 0.029].

A study conducted among North Carolina children states that, antibiotic use by children in the past 6 months were associated with nasal colonization of *S. aureus*. In addition to that, the study also reveals that, parental employment in a school/daycare and family history of boils were associated with *S. aureus* nasal carriage [9]. In line with that, our study also obtained family member with wound in the previous 12 months were associated with nasal colonization of *S. aureus* in the bivariate logistic regression analysis.

Concerning about recurrent AOM in the previous 12 months, a study conducted in Ethiopia by the corresponding author of this research (Alemayehu Reta) shows statistically significant association among school children [8].

In agreement with our finding, a study conducted in Nepal by Rijal KR and his colleagues illustrates that, children admitted to hospital in the past 12 months were statistically associated with nasal colonization of *S. aureus* [12] and also a study in Turkey children shows a similar finding, which is conducted among children having the age of 6 months up to 15 years, hospitalization or surgical operation in the previous 1 year were associated with *S. aureus* colonization [18] obviously, there are infectious agents in the hospital environment that can be considered as hospital acquired infection. *S. aureus* is one of the nosocomial infectious agent that can disseminate to the community when an admitted patient is discharged. So if the child is admitted previously, there will be a greater probability of being colonized by *S. aureus*.

## Conclusions and recommendations

This is the first study among Ethiopian pre-school children below 6 years of age studying nasal colonization of *S. aureus* and antimicrobial susceptibility pattern of the isolates and showed a relatively low prevalence of *S. aureus*. Most of the isolates were resistant to β-lactam drugs and sensitive to drugs like Gentamycin, Erythromycin, Clindamycin, Chloramphenicol, Doxycycline, Tetracycline, Kanamycin and Augmentin. The study shows that attending pre-school was associated with nasal colonization of *S. aureus*. Studies with cohort design plus molecular analysis of the isolates are needed to accurately assess the epidemiology of *S. aureus* nasal colonization in various geographical locations, to see the strain distribution and to detect heteroresistant isolates. More studies on additional associated factors with a large sample size, which accompanies various geographical locations should be done for a better understanding of *S. aureus* nasal colonization and to design preventive strategies that target bacterial colonization in the upper respiratory tract (throat, nose…).

## Abbreviations

AOM: acute otitis media
AOR: adjusted odds ratio
ATCC: American Type Culture Collection
CI: confidence interval
CLSI: Clinical and Laboratory Standard Institute
IQR: inter quartile range
MIC: minimum inhibitory concentration
MRSA: methicillin resistant *Staphylococcus aureus*
MSA: mannitol salt agar
SPSS: Statistical Package for the Social Science
